# Genome-wide identification and expression analysis of the CCT gene family reveals its function in regulating heading date in proso millet (*Panicum miliaceum* L.)

**DOI:** 10.3389/fpls.2026.1721307

**Published:** 2026-03-17

**Authors:** Qi Tan, Yanan Liu, Haotian Li, Li Dong, Guoqing Liu, Haiquan Li, Yanmiao Jiang

**Affiliations:** 1 Institute of Millet Crops, Hebei Academy of Agriculture and Forestry Sciences, Shijiazhuang, Hebei, China; 2 Key Laboratory of Minor Crops in Hebei, Shijiazhuang, Hebei, China

**Keywords:** CCT gene family, gene expression, genome-wide identification, heading date, photoperiod, proso millet

## Abstract

**Introduction:**

Proso millet (*Panicum miliaceum* L.) is an important short-day cereal crop whose heading date is highly sensitive to photoperiod, a key factor influencing its yield and geographical adaptation. The CCT (CONSTANS, CONSTANS-LIKE and TOC1) gene family plays a crucial role in regulating plant photoperiod pathway and flowering time.

**Methods:**

In this study, a comprehensive genome-wide identification, characterization and expression analysis were performed of the CCT gene family in proso millet. CCT family members were identified, and their phylogenetic relationships, gene structures, conserved motifs, and duplication events were analyzed. Expression patterns were examined using RNA-seq data from leaves and shoot apical meristems under different photoperiod conditions, and further analyzed by qRT‑PCR across proso millet germplasms with varying heading dates.

**Results:**

A total of 74 *CCT* genes (*PmCCT1–PmCCT74*) were identified and found to be unevenly distributed across the 18 chromosomes. Phylogenetic analysis classified them into four major subfamilies: CONSTANS (COL)-like, CCT MOTIF FAMILY (CMF)-like, pseudo-response regulator (PRR)-like, and TIFY-like. Expression profiling revealed that 17 *PmCCT* genes exhibited differential expression under long-day (LD) and short-day (SD) conditions, with elevated transcript levels in both leaves and spikelets. Subsequent qRT-PCR analysis across proso millet germplasms with varying heading dates further identified ten marker *PmCCT* genes that were strongly induced during the heading transition under SD treatment, specifically in the photoperiod-sensitive accession. Their dynamic expression patterns closely correlated with heading time variation.

**Discussion:**

This study provides a systematic understanding of the potential functions of *PmCCT* genes in photoperiod-mediated regulation of heading date in proso millet. The identified ten candidate marker genes highlight their role in photoperiod-dependent regulation, offering valuable molecular targets and a theoretical foundation for molecular breeding aimed at improving geographical adaptations of proso millet accessions.

## 1 Introduction

Proso millet (*Panicum miliaceum* L.) is a highly nutritious food crop in arid and semi-arid regions of northern China (Chen et al., 2023). Heading date is a key agronomic trait that determines crop yield, quality, and geographical adaptability. Its regulation largely depends on the plant’s ability to perceive and respond to photoperiod. As a typical short-day plant, proso millet is highly sensitive to photoperiod changes. Its heading date adjusts more markedly under varying photoperiods than in crops like rice and foxtail millet (Dong et al., 2020).

The photoperiodic flowering pathway in plants is a highly conserved and complex regulatory network, in which the CCT gene family serves as a key component. Based on the functional domains of the encoded proteins, members of the CCT gene family can be classified into four subfamilies: the CMF family, the COL family, the PRR family, and TIFY family (Huang et al., 2024; Li et al., 2022a). The CMF subfamily contains only a single CCT domain. The COL subfamily possesses one or more B-box zinc finger domains (including type I, type II, or multiple B-box zinc finger domains) along with one CCT domain. The PRR subfamily is characterized by the presence of a PRR domain and one CCT domain (Cockram et al., 2012; Jin et al., 2018). The CCT domain was originally described as a 43 amino acid region of homology found in the proteins CO (CONSTANS), CO-LIKE and TOC1 (TIMING OF CAB1), which have been well characterized as regulators of flowering in *Arabidopsis thaliana* (Putterill et al., 1995; Robson et al., 2001; Strayer et al., 2000).

Extensive studies on flowering time have been conducted in *Arabidopsis* and rice. *CCT* genes play important roles in the molecular regulatory network controlling flowering time. *FLOWERING LOCUS T (FT*) acts as a central integrator in the flowering pathway, encoding the mobile florigen signal in leaves (Abe et al., 2005). In *Arabidopsis*, *FT* transcription is activated by CO, which in turn is activated by Gigantea (GI) under LD conditions (Hayama and Coupland, 2003; Hayama et al., 2003). In rice, *Hd1* (*Heading date 1*, also known as *OsCCT21*), a homolog of *CO*, was the first cloned gene regulating heading date. *Hd1* was initially identified as a flowering activator under SD conditions and a repressor under LD conditions. Several other *CCT* family genes, including *Ghd7*, *Ghd7.1* (*OsCCT28*), *Ghd2* (*OsCCT9*), and *OsCCT19*, inhibit flowering under LD conditions (Liu et al., 2016b, Liu et al., 2013; Xue et al., 2008; Yan et al., 2013; Yano et al., 2000; Zhang et al., 2015). Additionally, some *CCT* genes, such as *OsCCT01*, *OsCOL4* (*OsCCT6*), *OsCOL10* (*OsCCT14*), *OsCOL13* (*OsCCT27*), *OsCOL15* (*OsCCT30*), and *OsNRRa* (*OsCCT18*), delay heading under both LD and SD conditions in rice (Lee et al., 2010; Liu et al., 2016a; Sheng et al., 2016; Tan et al., 2016; Wu et al., 2018, Wu et al., 2013). Additionally, the CCT gene family regulates biological processes in plants, including growth and development, and stress resistance (Cai et al., 2024; Yu et al., 2024; Zhang et al., 2024).

In recent years, genome-wide identification and systematic analysis of the CCT gene family have been conducted in various plant species, including rice (Zhang et al., 2021), foxtail millet (Li et al., 2022a), *Liriodendron chinense* (Zhu et al., 2025), and pear (Liu et al., 2022). These studies have elucidated the evolutionary patterns, expression characteristics, and pivotal roles of *CCT* genes in photoperiodic responses. However, research on the CCT gene family in proso millet has not yet been reported, leaving a gap in the systematic analysis of its potential expansion patterns and unique regulatory mechanisms. The shoot apical meristem (SAM) serves as the initiation site for panicle development, while spikelets are the subsequent reproductive structures formed through further differentiation (Chavez-Hernandez et al., 2022; Kanrar et al., 2008; Kaufmann et al., 2010). Monitoring the expression changes of *PmCCT* genes throughout this continuous process helps to identify key genes that simultaneously respond to photoperiod and regulate panicle development.

As an allotetraploid crop, proso millet has experienced genome duplication during evolution. Compared to diploid relatives such as foxtail millet and rice, this distinctive genomic background may lead to greater complexity and diversity in copy number, structural variation, and regulatory functions of its CCT gene family, making it an ideal system for studying gene family evolution and functional differentiation (Chen et al., 2023; Zou et al., 2019). This study identified 74 *PmCCT* genes through genome-wide analysis. Its novelty lies in an integrated methodological approach, which for the first time combines transcriptomic data from leaves and SAM under SD conditions with multi-tissue expression profiles. The analysis encompassed both photoperiod-sensitive and photoperiod-insensitive proso millet germplasms, with detection performed via qRT-PCR. This systematic strategy enabled the identification of ten *PmCCT* genes as candidate marker genes associated with photoperiod-regulated heading. By screening light-responsive tissues and key developmental stages, the study successfully identified these co-expressed regulatory genes.

The findings will enhance our understanding of the photoperiod response mechanisms in proso millet, particularly providing new insights into the evolutionary relationship between gene family expansion and the regulation of key agronomic traits in polyploid crops. Moreover, this study will provide valuable genetic resources for optimizing heading date and enhancing ecological adaptability via molecular breeding approaches.

## 2 Materials and methods

### 2.1 Plant materials and growth conditions

This study selected two photoperiod-insensitive proso millet germplasm accessions, 0016 and 0053, with a photoperiod-sensitive proso millet cultivar Longmi4 serving as the control. All seeds were obtained from the germplasm resource bank previously established by the Institute of Millet Crops, Hebei Academy of Agriculture and Forestry Sciences (Shijiazhuang, China).

The experiment was conducted in an artificial climate chamber under controlled conditions at 28 °C temperature and 60% humidity (Cong et al., 2025; Mushtaq et al., 2023). The normal long-day (LD) condition was 18 h light/6 h dark, and the SD treatment was 12 h light/12 h dark (Dong et al., 2020). Light intensity, temperature, and humidity were maintained consistent between different photoperiod regimes.

### 2.2 Identification and chromosome mapping of the CCT family members in proso millet

Genome and annotation data for proso millet (accession number GWHAAEZ00000000.1) were downloaded from the Genome Warehouse in the BIG Data Center (http://bigd.big.ac.cn/gwh). Genomic information, annotation data, and protein sequences for *Arabidopsis*, rice, and foxtail millet were obtained from the EnsemblPlants database (https://plants.ensembl.org/index.html). The Hidden Markov Model (HMM) program HMMER was used to identify CCT proteins with a domain e-value cutoff of <1×10^-14^. The HMMER profile of the CCT domain (PF06203) was downloaded from the PFAM database (http://xfam.org), and TBtools software was used to search for and identify CCT family members in the proso millet genome (Chen et al., 2020). All candidate sequences were submitted to the NCBI Conserved Domains Database (CDD) (http://www.ncbi.nlm.nih.gov/cdd/) for domain verification. Only sequences containing full-length CCT domains were considered as PmCCT proteins and used for further analysis. The names and corresponding amino acid sequences of the PmCCT family genes are listed in **Supplementary Table 1**.

Basic properties of the CCT proteins, including amino acid length, isoelectric point (pI), and molecular weight (MW), were estimated using the online tool ProtParam (http://web.expasy.org/protparam/). The identified *PmCCT* genes were mapped to proso millet chromosomes using TBtools software, and a chromosome location map was generated.

### 2.3 Phylogenetic evolution, gene structure and conserved motifs analysis

To understand the phylogenetic relationships and classification of CCT family genes, a phylogenetic tree was constructed based on the full-length amino acid sequences of CCT proteins from proso millet, *Arabidopsis*, foxtail millet, and rice. Multiple sequence alignment was performed using MUSCLE. The MEGA11 software was employed to construct the phylogenetic tree by the Neighbor-Joining (NJ) method with 1000 bootstrap replicates for statistical reliability. The evolutionary tree was visualized and annotated using iTOL (https://itol.embl.de). The MEME online tool was used to identify conserved motifs in PmCCT proteins, with the maximum number of motifs set to 10 (Bailey et al., 2015).

### 2.4 Analysis of CCT family genes duplication events

The intra-specific duplication events of CCT family genes in proso millet were analyzed using the Advanced Circos feature in TBtools. Inter-species collinearity analysis of CCT family genes among proso millet, *Arabidopsis*, rice, and foxtail millet was conducted using the One-Step MCScanX feature in TBtools. The values of Ks, Ka, and Ka/Ks were calculated using both MEGA11 and TBtools.

### 2.5 RNA extraction

Total RNA was extracted from proso millet leaves or SAM using FreeZol Reagent (Vazyme, R711-01) and reverse transcribed into cDNA using HiScript III RT SuperMix (Vazyme, R323‐01).

### 2.6 Expression pattern analysis

This study comprised three distinct expression analyses: (1) tissue-specific expression profiling of the *PmCCT* genes using public RNA-seq data; (2) transcriptome analysis of leaves and SAM in Longmi4 under SD conditions, followed by qRT-PCR validation; and (3) qRT-PCR analysis of *PmCCT* genes expression before and after heading in different proso millet accessions under SD treatment.

(1) Analysis of the expression level of the *PmCCT* genes in different tissues of proso millet was performed using publicly available RNA-seq data. To investigate the expression patterns of the *PmCCT* genes, this study utilized a previously published RNA-seq dataset. The experimental material used was a proso millet landrace (accession number: 00000390), covering ten tissues: root, stem, leaf, seedling, seed, panicle, leaf sheath, and spikelet (Zou et al., 2019). Expression levels of *PmCCT* genes were quantified based on FPKM values and extracted entirely from this database.
(2) Transcriptome analysis of leaves and SAM in Longmi4 under SD treatment and qRT-PCR validation. This experiment aimed to elucidate transcriptomic changes in the *PmCCT* genes induced by SD treatment. The photoperiod-sensitive cultivar Longmi4 was used as the experimental material. After sowing, seedlings were initially cultivated under LD conditions for three weeks, followed by transfer to SD conditions for treatment. The tips of the youngest fully expanded leaves and the SAM of proso millet were separately collected. Sampling time points were set before treatment (SD 0-day: S0) and on the 5th (SD 5-day: S5) and 10th (SD-10 day: S10) days after treatment. For each tissue at each time point, three independent biological replicates were established. Samples were immediately flash-frozen in liquid nitrogen after collection and stored at -80 °C for subsequent use. RNA-seq analysis was conducted on these samples. To validate the reliability of the RNA-seq data, qRT-PCR analysis was performed on the same RNA samples used for transcriptome sequencing. These samples included leaves and SAM of Longmi4 collected at S0, S5, and S10. A total of 17 candidate *PmCCT* genes were selected for validation, their expression patterns were compared with the RNA-seq analysis results.
(3) qRT−PCR analysis of *PmCCTs* expression in proso millet under SD conditions. This study analyzed the expression patterns of *PmCCT* genes before and after heading in different proso millet accessions under SD treatment by qRT−PCR. Proso millet accessions with contrasting photoperiod sensitivities were selected for the experiment, including the photoperiod−sensitive accession Longmi4 and the photoperiod−insensitive accessions 0016 and 0053. All materials were cultivated under continuous SD conditions from seed germination onward, and their heading dates were recorded. Under continuous SD conditions, the photoperiod-sensitive proso millet cultivar Longmi4 and the photoperiod-insensitive accessions 0016 and 0053 remained non headed at 21 days. By day 28, early heading was observed in Longmi4, whereas accessions 0016 and 0053 still showed no heading response. To further investigate the expression of *PmCCT* genes, the tips of the youngest fully expanded leaves were collected from plants of each accession at 21-day and 28-day after sowing. Three biological replicates were included for each accession and each time point. All collected samples were immediately flash−frozen in liquid nitrogen and stored at -80 °C until use. Subsequent experiments involved total RNA extraction, reverse transcription, and qRT−PCR analysis to examine changes in target *PmCCT* genes expression before and after heading.

Integrated analysis of photoperiod-responsive and tissue−specific expression. An integrated analysis was performed to identify *PmCCT* genes that are both responsive to photoperiod and highly expressed in tissues relevant to heading. For this purpose, the RNA−seq data generated in this study (from leaves and SAM of Longmi4 under SD) were combined with the public multi−tissue expression dataset from Zou et al. (2019).

First, the *PmCCT* genes that were differentially expressed in the SD−treated leaves or SAM were selected from the RNA-seq dataset in this study. Separately, to identify genes with tissue-specific expression, the public multi-tissue transcriptome dataset (Zou et al., 2019), comprising ten distinct tissues, was analyzed. For each *PmCCT* gene, expression levels across all ten tissues were ranked. Genes were considered to exhibit “high expression in leaves” if leaf tissue ranked among the top three across all tissues; similarly, “high expression in spikelets” was defined if spikelet tissue ranked among the top three. The union of these two independent sets was then identified, yielding candidate genes that were considered both photoperiod−responsive and highly expressed in heading−related tissues.

### 2.7 Transcriptome and qRT-PCR analysis

RNA-seq was performed on the Illumina NovaSeq 6000 platform by Biomarker Technologies (Qingdao, China). Subsequent bioinformatic data analysis was conducted via the Biomarker Cloud Platform (https://www.biocloud.net/). The workflow included: alignment of quality-controlled sequencing reads to the proso millet reference genome (*Panicum miliaceum* Longmi_v2) using HISAT2 (v3.0.3); transcript assembly and gene expression quantification (FPKM) using StringTie; and finally, differential expression analysis using DESeq2, with screening thresholds set at |log_2_FC| > 1 and FDR < 0.05.

The qRT-PCR was performed on a Bio-Rad CFX96 system using ChamQ Universal SYBR qPCR Master Mix (Vazyme, Q711-03). The 20 μL reaction mixture contained 10 μL of 2×SYBR mix, 0.4 μL of each primer (10 μM), 2 μL of cDNA, and 7.2 μL of nuclease-free water. The thermal cycling protocol was: 95°C for 30 s; 40 cycles of 95°C for 10 s and 60°C for 30 s.

Gene expression levels were calculated using the 2^(-ΔΔCt) method with *PmActin* serving as the internal reference gene. For RNA-seq validation, expression was normalized to the Longmi4 sample at SD 0-day sample in leaf and SAM, respectively. For analysis of *PmCCTs* expression levels across different proso millet accessions, expression was normalized to the Longmi4 sample at SD 21 day. The qRT-PCR primers are listed in **Supplementary Table 2**.

## 3 Results

### 3.1 Identification and chromosome distribution of CCT gene family in proso millet

**Figure 1:**
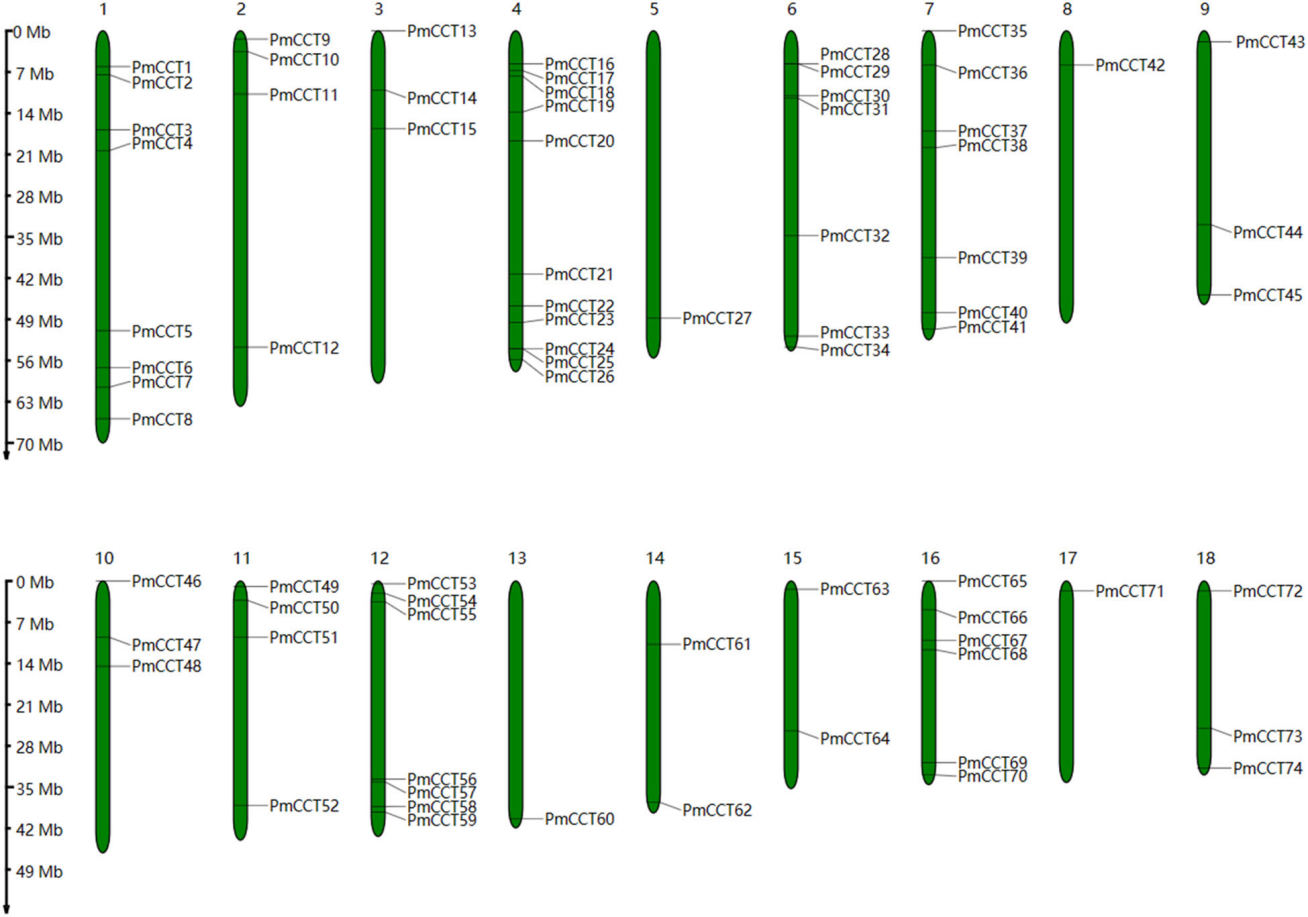
Chromosomal distribution of the CCT gene family in proso millet. The scale on the left indicates the base pair lengths (Mb) of the chromosomes.

A total of 74 non-redundant *PmCCT* genes were identified in proso millet. These genes were named *PmCCT1* to *PmCCT74* based on their chromosomal positions. Chromosome mapping analysis showed that the 74 *PmCCT* genes were unevenly distributed across the 18 chromosomes, with chromosome 4 containing the most members (11), while chromosomes 5, 8, 13, and 17 each contained only one member (**Figure 1**). The amino acid lengths of these CCT proteins varied considerably, ranging from 148 (PmCCT66) to 775 (PmCCT53), and the molecular weights ranged from 16.81 kDa (PmCCT66) to 84.13 kDa (PmCCT23). The isoelectric points ranged from 4.2 (PmCCT19) to 11.7 (PmCCT66) (**Supplementary Table 3**).

### 3.2 Phylogenetic analysis of CCT gene family

**Figure 2:**
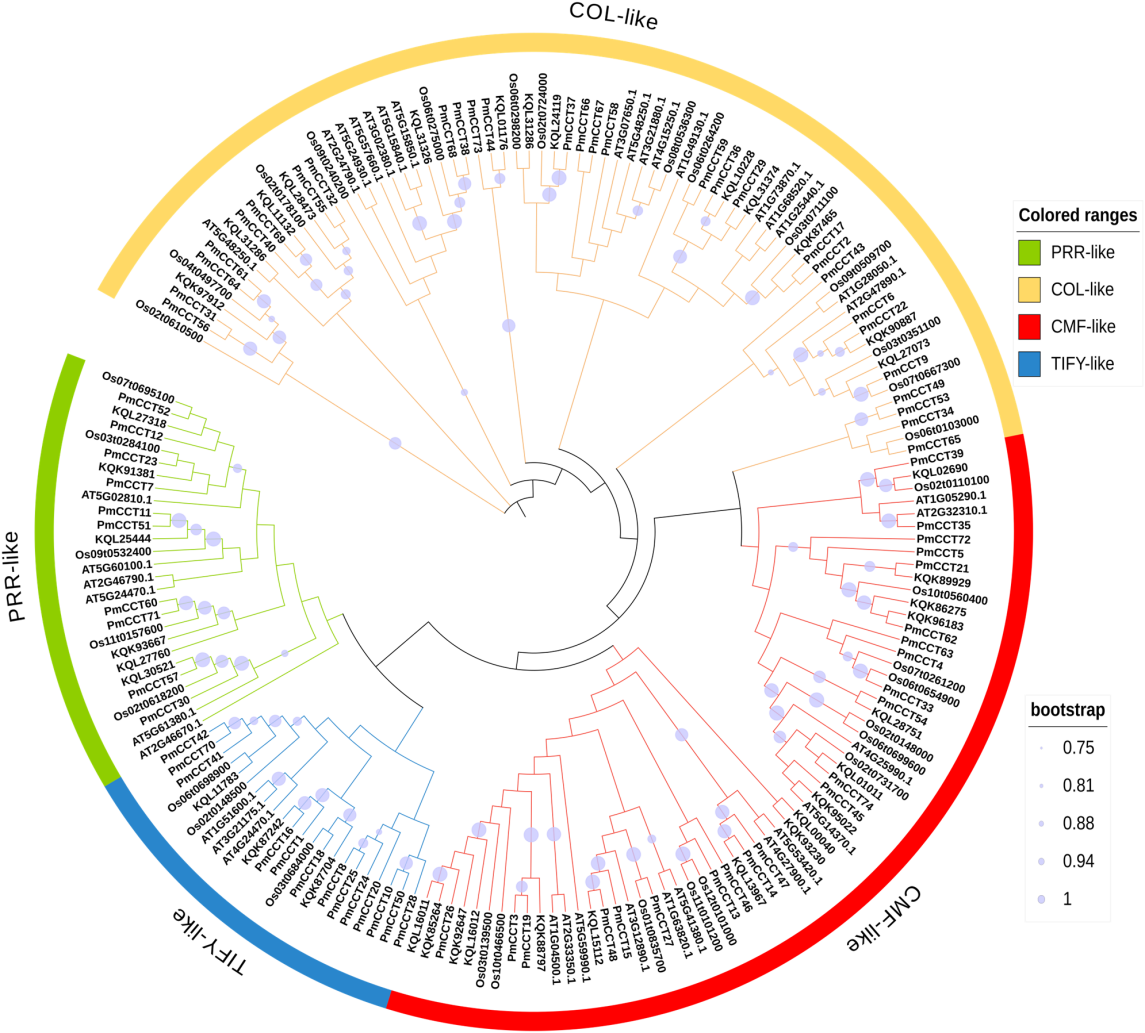
Phylogenetic analysis of CCT family genes. A NJ tree was constructed based on the CCT family genes identified from proso millet, *Arabidopsis*, rice, and foxtail millet. According to their evolutionary relationships, solid-line boxes in different colors delineate four major clades: COL-like, CMF-like, PRR-like, and TIFY-like subfamilies.

To elucidate the evolutionary relationships within the CCT gene family, the phylogenetic tree was constructed based on full-length CCT protein sequences, including 74 from proso millet, 39 from *Arabidopsis*, 34 from rice, and 37 from foxtail millet. The number of CCT family genes identified in rice and foxtail millet aligns closely with previously published data (Li et al., 2022a; Zhang et al., 2021). Based on the domain characteristics of the genes, all *CCT* genes can be classified into four subfamilies: the COL-like subfamily has the largest number, comprising 73 genes; the CMF-like subfamily follows, with 62 members; the PRR-like subfamily contains 27 members; and the TIFY-like subfamily consists of 22 members. Regarding the distribution of CCT family genes in proso millet, 29 of its members belong to the COL-like subfamily, 22 to the CMF-like subfamily, 13 to the TIFY-like subfamily, and 10 to the PRR-like subfamily. The distribution of these subfamilies across proso millet, *Arabidopsis*, foxtail millet, and rice is relatively balanced, indicating that the expansion patterns of *CCT* genes are similar among these species (**Figure 2**).

### 3.3 Gene structure and conserved motifs of PmCCT gene family

**Figure 3:**
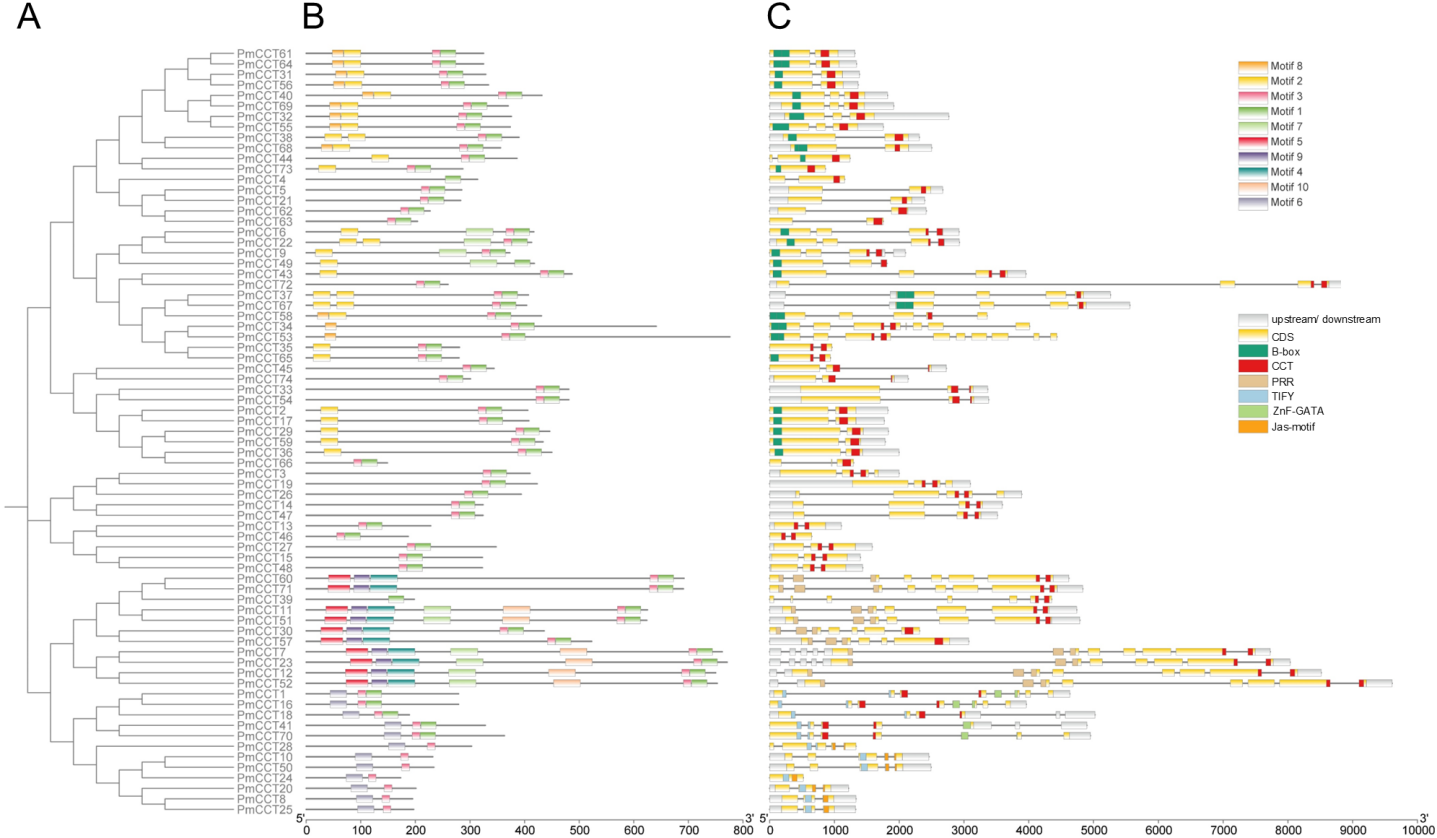
The phylogenetic tree, gene structure, and conserved motifs of the *PmCCT* family genes in proso millet. **(A)** A NJ phylogenetic tree was constructed based on the full-length protein sequences of PmCCTs. **(B)** Ten conserved motifs of the PmCCT gene family. Boxes of different colors represent distinct conserved motifs. **(C)** Schematic diagram of *PmCCT* genes structures. yellow boxes represent exons, black lines represent introns, gray boxes represent the flanking regions (upstream/downstream) of the gene, green boxes represent the B-box domain, red boxes represent the CCT domain, beige boxes represent the PRR domain, blue boxes represent the TIFY domain, light green boxes represent the ZnF-GATA domain, orange boxes represent the Jas-motif.

To systematically investigate the evolutionary relationships and structural characteristics of the PmCCT gene family in proso millet, a NJ phylogenetic tree was constructed based on the full-length protein sequences of PmCCT (**Figure 3A**). Subsequently, a comprehensive analysis of gene structures and conserved motifs was performed for the 74 family members.

Conserved motif analysis revealed a total of 10 conserved motifs within the PmCCT family. The distribution of these motifs exhibited clear phylogenetic clade specificity: members from different evolutionary branches displayed not only distinct motifs compositions but also characteristic combinations of protein domains. Among the 74 *PmCCT* genes, Motif 1, Motif 2, and Motif 5 showed partial overlap in their distribution, being present in 70, 27, and 10 genes, respectively (**Figure 3B**).

Gene structure analysis demonstrated that *PmCCT24* and *PmCCT73* completely lack introns, indicating a very low proportion of intron-less genes within this family. Half of the family members possess complete upstream and downstream flanking regions, while 29 genes lack a regulatory region on one side, and eight genes completely lack flanking regions on both sides. Notably, nearly all family members contain at least one of the following domains: CCT, B-box, ZnF, or TIFY (**Figure 3C**). These findings collectively reveal the complex domain architecture and motif distribution patterns within the PmCCT gene family, suggesting potential functional diversification among its members.

### 3.4 Duplication and collinearity analysis of the PmCCT gene family

The 74 *PmCCT* genes were mapped to their respective chromosomes and found to be irregularly distributed across the 18 chromosomes. To investigate the expansion mechanisms and gene duplication events within the PmCCT gene family, analyses of tandem and segmental duplications were performed. In the proso millet genome, a total of 58 duplicated *PmCCT* gene pairs were identified, including 56 segmentally duplicated pairs and 2 tandemly duplicated pairs. Tandem duplication events occurred primarily on chromosome 4. The Ka/Ks ratios for these duplicated gene pairs were all less than 1.00, indicating the action of purifying selection (**Supplementary Table 4**). These findings suggest that both tandemly and segmentally duplicated genes have undergone purifying selection, and that segmental duplication has played a critical role in the expansion of the CCT gene family in proso millet.

**Figure 4:**
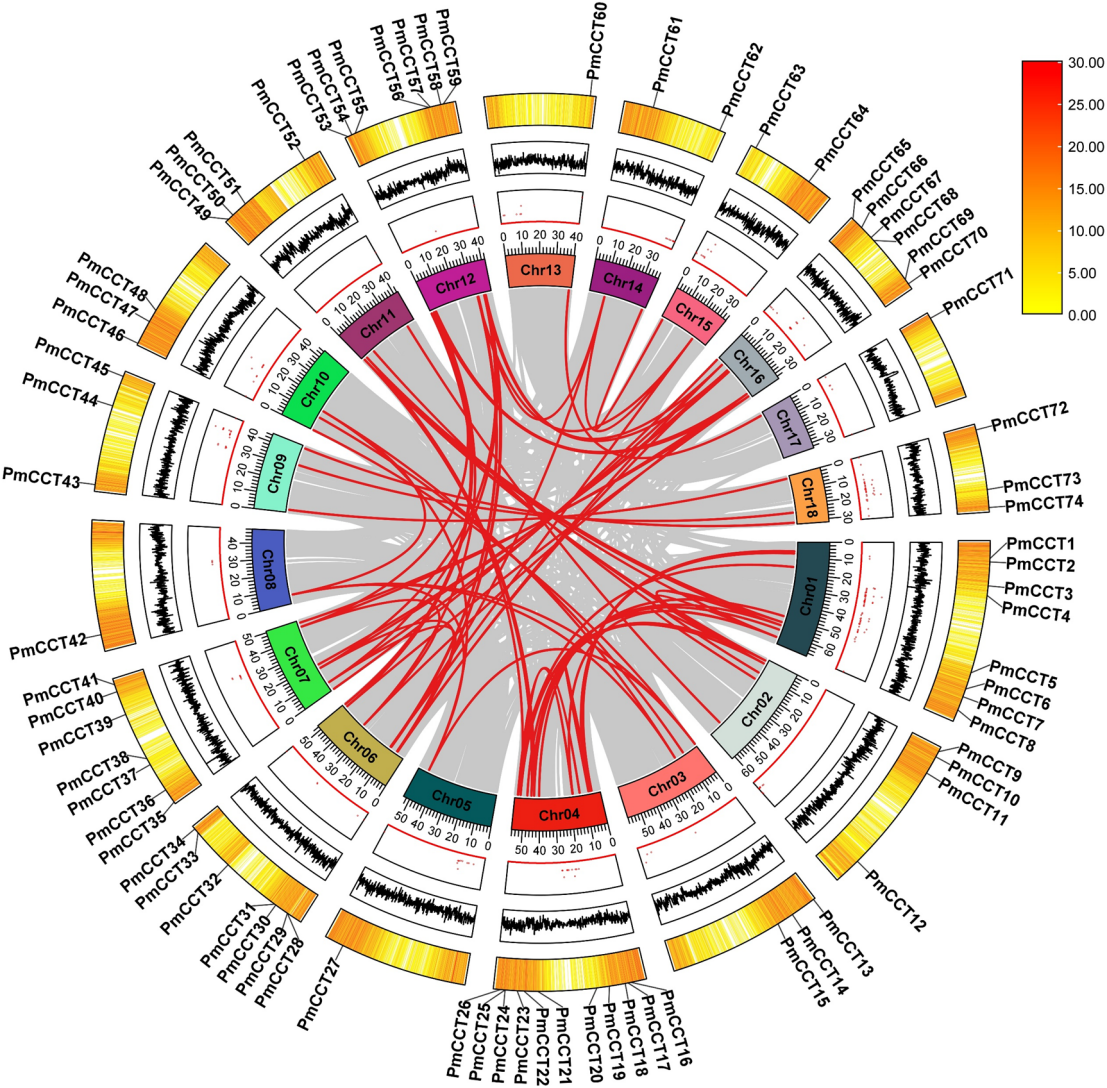
Intraspecies collinearity analysis of the PmCCT family genes. From the outermost to the innermost layers, the figure shows the gene density on chromosomes, the GC content, and the gap distribution in the genome. In the inner ring, red lines are used to connect collinear *PmCCT* gene pairs across the whole genome.

To elucidate the evolutionary history of the PmCCT gene family in proso millet, a systematic collinearity analysis was conducted. The results identified 78 collinear *PmCCT* gene pairs, with chromosome 6 showing the highest density (18 pairs) and chromosome 13 the fewest (only one pair). The collinearity relationships of *PmCCT* genes are illustrated in **Figure 4**.

### 3.5 Collinearity analysis of CCT gene family in proso millet and other species

**Figure 5:**
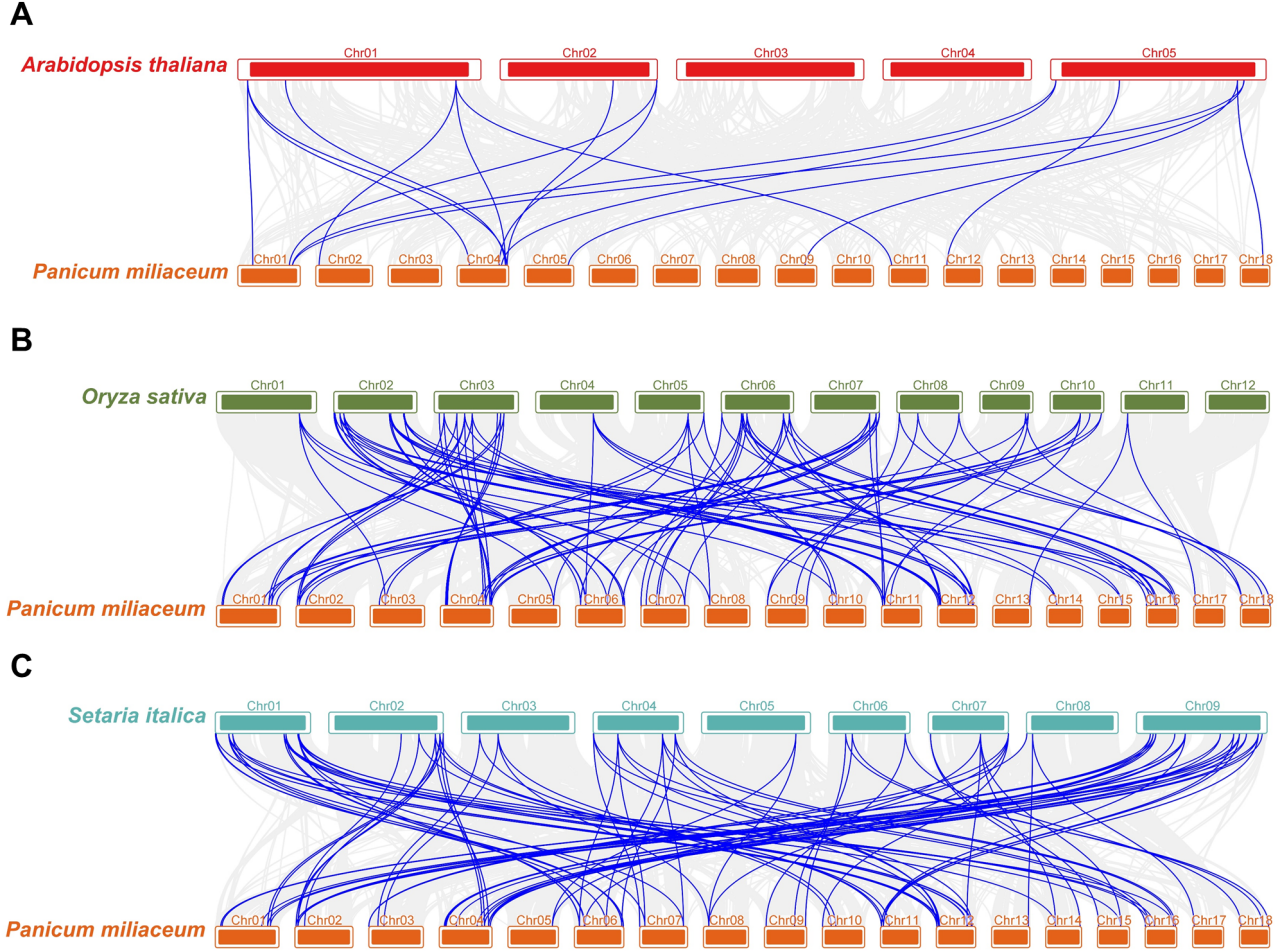
Interspecies collinearity analysis of CCT family genes between proso millet and other plant species. **(A)** Interspecies collinearity analysis of CCT family genes between proso millet and *Arabidopsis thaliana*; **(B)** Interspecies collinearity analysis of CCT family genes between proso millet and rice; **(C)** Interspecies collinearity analysis of CCT family genes between proso millet and foxtail millet. The gray lines represent collinear blocks between the proso millet genome and the other species, while the blue lines indicate collinearity among *CCT* genes.

To investigate the origin and evolutionary mechanisms of *PmCCT* genes, a comparative collinearity map was constructed between proso millet, *Arabidopsis*, rice, and foxtail millet. The results showed 17 orthologous gene pairs between proso millet and *Arabidopsis*, 164 orthologous gene pairs between proso millet and rice, and 175 orthologous gene pairs between proso millet and foxtail millet (**Figure 5**).

The analysis revealed multiple gene correspondences between proso millet and these three species, with homologous gene pairs categorized into three types: (1) one *PmCCT* gene in proso millet corresponding to one gene in other species; (2) one *PmCCT* gene in proso millet corresponding to multiple genes in other species; and (3) multiple *PmCCT* genes in proso millet corresponding to one gene in other species. The evolutionary relationship of the PmCCT gene family is relatively conserved with rice and foxtail millet, which also belong to the Poaceae family, but shows greater divergence with *Arabidopsis* from the Brassicaceae family.

### 3.6 Expression profile analysis of *PmCCT* genes

**Figure 6:**
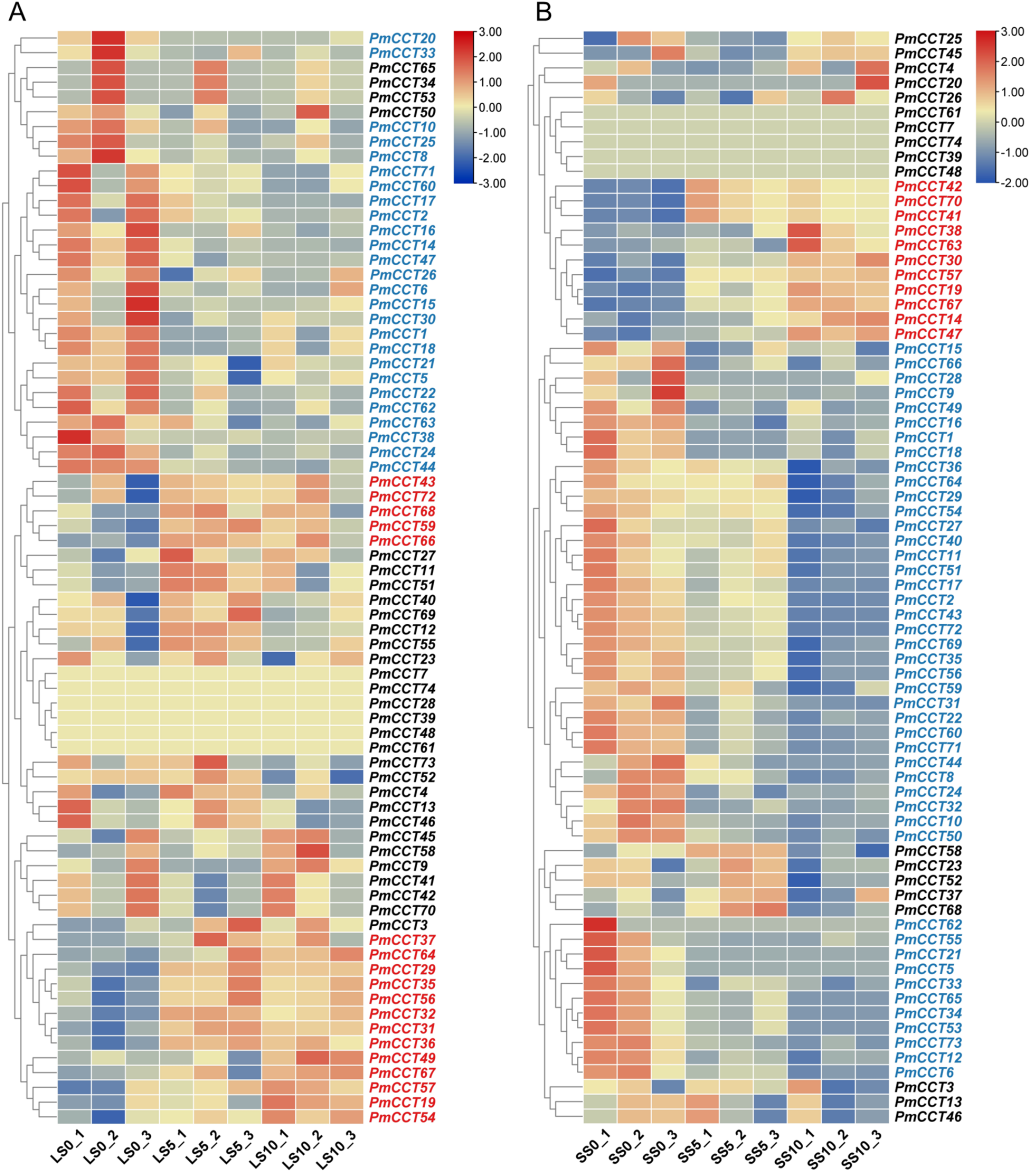
Analysis of expression patterns of *PmCCT* genes. **(A)** Heatmap analysis of transcriptomic data for *PmCCT* genes in leaves under conditions shifting from LD to SD photoperiods. LS0 represents the transcript level in leaves before SD treatment, LS5 represents the transcript level in leaves after 5 days of SD treatment, and LS10 represents the transcript level in leaves after 10 days of SD treatment. **(B)** Heatmap analysis of transcriptomic data for *PmCCT* genes in SAM under conditions shifting from LD to SD photoperiods. SS0 represents the transcript level in SAM before SD treatment, SS5 represents the transcript level in SAM after 5 days of SD treatment, and SS10 represents the transcript level in SAM after 10 days of SD treatment. Blue font indicates downregulated expression, and red font indicates upregulated expression.

To elucidate the expression response patterns of the *PmCCT* genes to photoperiod, transcriptomic data of leaves and SAM from Longmi4 under SD treatment were analyzed. The result showed that SD treatment induced differential expression of 44 *PmCCT* genes (genes exhibiting consistent up-regulated or down-regulated) in leaves among which 18 genes showed sustained upregulation (marked in red) and 26 genes showed sustained downregulation (marked in blue) (**Figure 6A**; **Supplementary Table 5**). In the SAM, SD treatment led to differential expression of 56 *PmCCT* genes, including 11 genes with sustained up-regulated and 45 genes with sustained down-regulated (**Figure 6B**; **Supplementary Table 5**). Additionally, six *PmCCT* genes (*PmCCT7*, *PmCCT28*, *PmCCT39*, *PmCCT48*, *PmCCT61*, and *PmCCT74*) exhibited no detectable expression in leaves under SD conditions. Five *PmCCT* genes (*PmCCT7*, *PmCCT39*, *PmCCT48*, *PmCCT61*, and *PmCCT74*) were also not expressed in the SAM (**Figures 6A, B**).

It is important to clarify that the SAM and spikelets represent two consecutive developmental stages in proso millet panicle formation. The SAM is the site where panicle initiation begins, while spikelets are reproductive structures formed later through SAM differentiation. Therefore, genes responding to photoperiod in the SAM may regulate the initiation of panicle development, whereas those highly expressed in spikelets are more likely involved in the later differentiation and maturation of floral organs. To capture both early and late regulatory events, transcriptomic data from SD-treated SAM (this study) and publicly available spikelet expression data (Zou et al., 2019) were integrated in subsequent screening.

**Figure 7:**
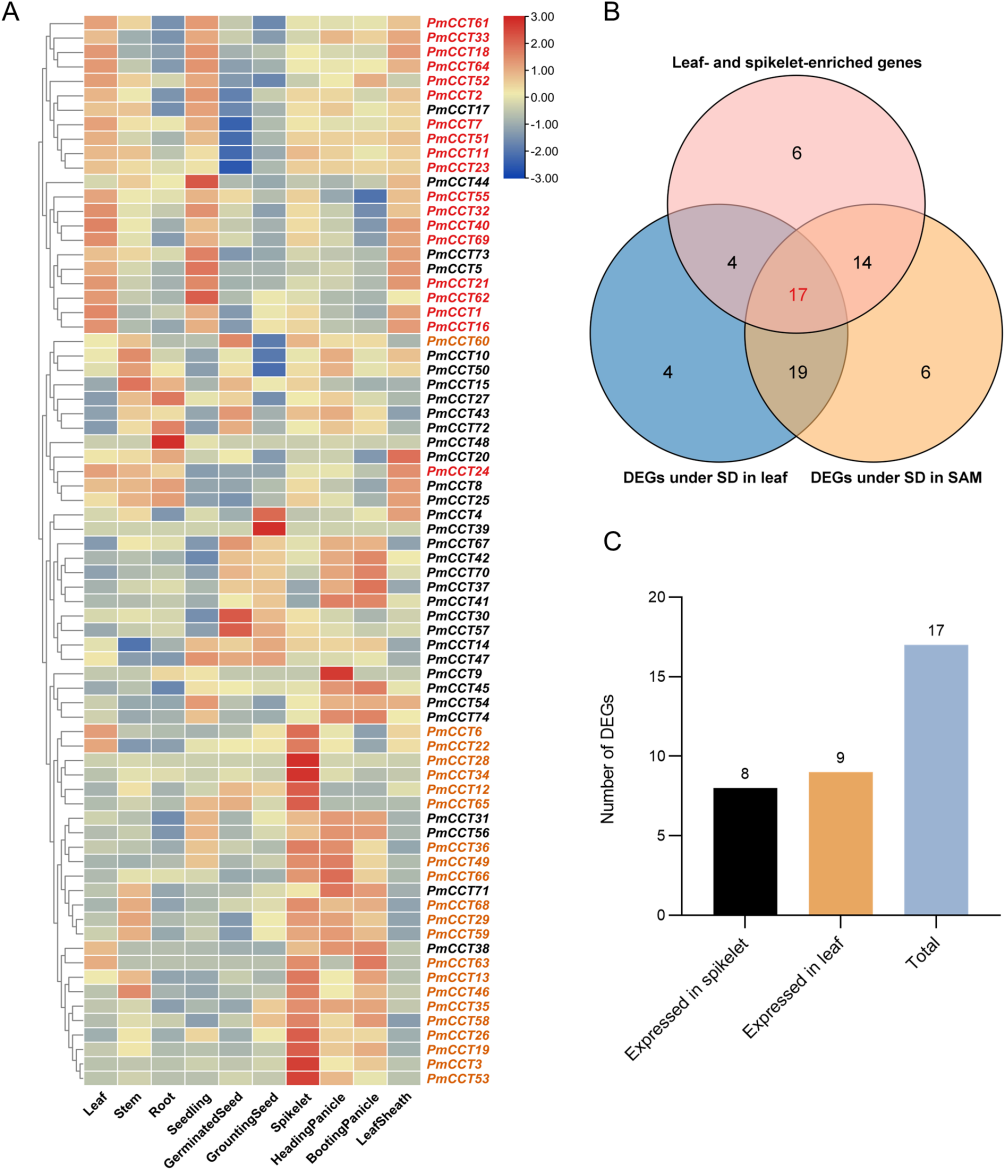
Tissue-specific analysis of candidate genes responsive to photoperiod changes in proso millet. **(A)** Heatmap showing transcript levels of *PmCCT* genes across ten tissues. Genes in red font indicate high expression in leaves (ranked in the top three across all tissues), and genes in orange font indicate high expression in spikelets (ranked in the top three across all tissues). **(B)** The Venn diagram illustrates the intersections and distribution among three sets of genes: differentially expressed genes in the SAM under SD conditions, differentially expressed genes in leaves under SD conditions, and highly expressed genes in leaves or spikelets. **(C)** Statistics of highly expressed genes in leaves and spikelet tissues that overlap with genes identified in **(B)**.

Based on this, *PmCCT* genes with tissue-specific high expression were further screened. Using the multi-tissue expression dataset, expression levels of each *PmCCT* gene were ranked across all ten tissues. Genes were selected if their expression in leaves ranked among the top three (designated as high expression in leaves) or if their expression in spikelets ranked among the top three (designated as high expression in spikelets). This initial screening yielded 19 genes highly expressed in leaves and 22 in spikelets (**Figure 7A**, **Supplementary Table 6**).

*PmCCT* genes meeting both of the following criteria were selected from the above gene sets: (1) exhibiting significant differential expression in leaf or SAM in response to photoperiod treatment; and (2) showing high expression in leaves or spikelets. Through this intersection screening, 17 differentially expressed candidate genes were identified, including: *PmCCT1*, *PmCCT2*, *PmCCT6*, *PmCCT16*, *PmCCT18*, *PmCCT19*, *PmCCT21*, *PmCCT22*, *PmCCT24*, *PmCCT29*, *PmCCT32*, *PmCCT35*, *PmCCT36*, *PmCCT59*, *PmCCT62*, *PmCCT63*, and *PmCCT64* (**Figure 7B**). Further expression profiling analysis indicated that 8 of these genes exhibited the highest expression abundance in spikelets, while the remaining 9 genes showed peak expression abundance in leaves (**Figure 7C**). The *PmCCT* genes induced by photoperiod in leaves are likely involved in photoperiod sensing and signaling, whereas those responsive in the SAM may directly regulate the initiation of panicle development. By integrating these two aspects, 17 *PmCCT* genes were identified as key candidates linking photoperiod signals to the heading phenotype in proso millet. These candidate genes may therefore play crucial roles in the perception and response to photoperiod signals, as well as in the regulation of heading processes.

### 3.7 Validation of RNA-seq data by qRT-PCR

To confirm the accuracy of the transcriptomic profiles, qRT-PCR analysis was performed on the 17 candidate *PmCCT* genes using the identical RNA samples that were subjected to RNA-seq (leaves and SAM of Longmi4 under SD treatment at S0, S5, and S10). In both leaf and SAM tissues, all 17 genes exhibited expression trends consistent with the RNA-seq data (**Supplementary Figure 1** A–Q for leaves; **Supplementary Figure 2** A–Q for SAM). The validation results from qRT-PCR confirmed the robustness and reliability of the RNA-seq data obtained in this study, thereby providing a solid foundation for subsequent analyses.

### 3.8 Identification of *PmCCT* marker genes associated with heading transition under SD conditions

**Figure 8:**
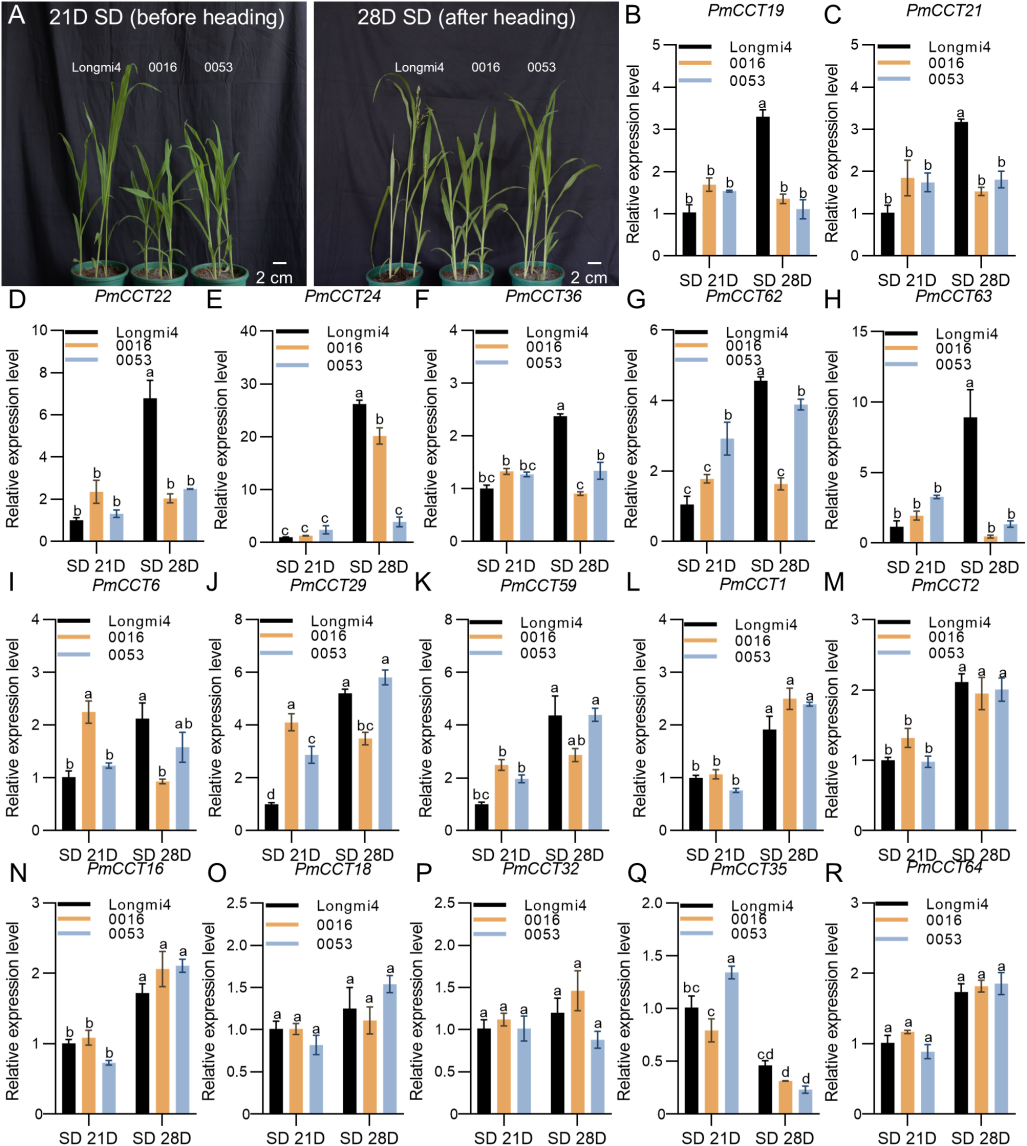
Identification and expression profiling of key photoperiod-responsive *PmCCT* genes. **(A)** Phenotypes of Longmi4 (headed), 0016, and 0053 (unheaded) after 21 and 28 days of SD treatment. **(B–R)** Transcript levels of the 17 candidate *PmCCT* genes in Longmi4, 0016, and 0053 before and after the heading of Longmi4 in **(A)**. Bar heights represent the mean expression levels from three independent biological replicates, and error bars indicate the standard error of the mean (SEM). All experiments were repeated at least three times with similar results. Different letters above the columns indicate significant differences as determined by one‐way analysis of variance (*P* < 0.05).

To further identify the functions of the 17 genes, during the screening of germplasm accessions, we identified two accessions, 0016 and 0053, that exhibited significantly delayed heading under SD conditions compared to the photoperiod-sensitive accession Longmi4 (**Figure 8A**). qRT-PCR analysis of materials subjected to SD treatment for 21 and 28 days revealed distinct expression dynamics among the 17 candidate genes. Based on their expression patterns, these genes were classified into three groups.

The first group comprised seven genes—*PmCCT19*, *PmCCT21*, *PmCCT22*, *PmCCT24*, *PmCCT36*, *PmCCT62*, and *PmCCT63*—which exhibited significantly higher expression levels in Longmi4 at day 28 compared to the photoperiod-insensitive accessions 0016 and 0053 (**Figures 8B–H**). Notably, these genes also showed a marked increase in expression from day 21 to day 28 specifically in Longmi4, coinciding with its heading transition.

The second group included *PmCCT6*, *PmCCT29*, and *PmCCT59*. Although these genes did not show significantly higher absolute expression in Longmi4 at day 28, they displayed a striking expression pattern: their transcript levels increased substantially from day 21 to day 28 in Longmi4, but remained unchanged or decreased in 0016 and 0053 over the same period (**Figures 8I–K**). This dynamic upregulation specifically in the heading-sensitive material during the heading transition suggests a potential association with the heading process.

The remaining seven genes (*PmCCT1*, *PmCCT2*, *PmCCT16*, *PmCCT18*, *PmCCT32*, *PmCCT35*, and *PmCCT64*) showed no consistent differences or dynamic changes among the three accessions at either time point (**Figures 8L–R**).

Through transcriptomic analysis and qRT-PCR analysis, ten *PmCCT* genes (*PmCCT6*, *PmCCT19*, *PmCCT21*, *PmCCT22*, *PmCCT24*, *PmCCT29*, *PmCCT36*, *PmCCT59*, *PmCCT62*, and *PmCCT63*) were identified as candidate marker genes associated with heading transition in proso millet. Their dynamic expression patterns, characterized by specific upregulation during the heading period in the photoperiod-sensitive accession Longmi4, suggest they may serve as molecular markers for photoperiod sensitivity and heading date variation.

## 4 Discussion

Proso millet, an ancient crop originating from China, possesses not only unique nutritional value but also serves as an important model species for studying photoperiod responses (Habiyaremye et al., 2016). Previous studies have demonstrated that the CCT gene family plays critical roles in regulating flowering, development, and circadian rhythms in plants. However, the specific functions of *PmCCT* genes in mediating heading date through photoperiod response in proso millet remain completely unknown. Therefore, this study presents the first genome-wide systematic analysis of the CCT gene family in proso millet, aiming to elucidate its role in photoperiod-regulated heading.

### 4.1 Significant expansion of the PmCCT gene family in proso millet

A total of 74 *PmCCT* genes were identified, unevenly distributed across all chromosomes of proso millet. The number of *PmCCT* genes in proso millet is significantly higher than in *Arabidopsis*, rice, and foxtail millet, and also exceeds that in diploid species such as tulip tree and pear (Li et al., 2022a; Liu et al., 2022; Zhang et al., 2021; Zhu et al., 2025). This is likely related to proso millet being an allotetraploid (Hunt et al., 2014). Although the ancestral species of proso millet remain unclear, two highly divergent ancestors may have provided a richer source of genetic variation, potentially endowing the *PmCCT* gene family with more diverse functional capabilities (Chen et al., 2023; Lu et al., 2024).

### 4.2 Segmental duplication is the primary driver of PmCCT gene family expansion

Whole-genome duplication, tandem duplication, and segmental duplication are major drivers of gene family expansion and functional differentiation. A total of 58 duplication events were identified within the PmCCT gene family, of which 56 were classified as segmental duplications, indicating that segmental duplication is the predominant mechanism driving the expansion of the PmCCT gene family. This finding is consistent with observations in foxtail millet (Li et al., 2022b). The prevalence of segmental duplications across diverse species suggests a conserved evolutionary pattern for the CCT family, likely linked to its essential roles in fundamental biological processes such as photoperiod response.

All duplicated gene pairs exhibited Ka/Ks ratios below 1.00, indicating that they have undergone strong purifying selection and functional constraint. This result aligns with findings in Chinese cabbage (Liu et al., 2025), foxtail millet (Li et al., 2022b), and soybean (Yu et al., 2024). Intraspecific collinearity analysis in proso millet revealed the highest density of collinear gene pairs on chromosome 6, suggesting that this chromosomal region may serve as a key region for PmCCT gene family duplication and retention.

### 4.3 Conserved synteny and lineage-specific expansion of CCT genes between proso millet and foxtail millet

The 74 PmCCT genes were unevenly distributed across all 18 chromosomes of proso millet, with notable enrichment in telomeric regions. Intraspecific collinearity analysis showed the highest density of collinear gene pairs on chromosome 4. Cross-species comparative analysis revealed that proso millet shares 17, 164, and 175 syntenic gene pairs with *Arabidopsis*, rice, and foxtail millet, respectively. The increasing number of syntenic pairs reflects closer phylogenetic relationships, consistent with the principle that species with closer evolutionary relatedness exhibit higher genomic collinearity (Xu et al., 2024).

Proso millet and foxtail millet exhibited the most extensive synteny, indicating high functional similarity between their *CCT* gene members. This result is consistent with the overall evolutionary framework of the CCT gene family in Poaceae, in which the four major subfamilies originated prior to the monocot-dicot divergence and subsequently experienced ancient whole-genome duplication events (Chen et al., 2023; Li et al., 2022a; Zou et al., 2019). The extensive collinearity observed between proso millet and foxtail millet reflects the legacy of these ancient genomic events following recent speciation.

The significantly larger number of *CCT* genes in proso millet compared to foxtail millet is likely attributable to its allotetraploid nature (Hunt et al., 2014). The allopolyploidy of proso millet provides a compelling explanation for its larger CCT gene repertoire relative to its diploid relative foxtail millet, and this lineage-specific expansion implies that *PmCCT* genes may have acquired more diverse or specialized functions.

### 4.4 *PmCCT* genes exhibit tissue-specific expression patterns in response to photoperiod

Integration of RNA-seq data identified 17 *PmCCT* genes that were highly expressed in both leaves and spikelets and exhibited differential expression under varying photoperiod conditions. Tissue- and developmental stage-specific expression of *CCT* genes has been documented in multiple species (Huang et al., 2024; Liu et al., 2025), and their high expression in leaves is consistent with their molecular functions in regulating photosynthesis and flowering (Liu et al., 2020).

By integrating RNA-seq data from proso millet under SD treatment and across different tissue types, this study identified 17 *PmCCT* genes that were highly expressed in both leaves and spikelets, and showed differential expression under varying photoperiod conditions. Among them, *PmCCT1*, *PmCCT2*, *PmCCT6*, *PmCCT16*, *PmCCT18*, *PmCCT21*, *PmCCT22*, *PmCCT24*, and *PmCCT62* were consistently downregulated in both leaves and SAM, while *PmCCT19* was consistently upregulated in both tissues. Additionally, several *PmCCT* genes showed completely opposite expression patterns in leaves and SAM, such as *PmCCT29*, *PmCCT32*, *PmCCT35*, *PmCCT36*, *PmCCT59*, *PmCCT63*, and *PmCCT64*. Furthermore, the reliability of the transcriptomic data in this study was confirmed through qRT-PCR validation using the same set of samples, where all 17 genes in both leaves and SAM showed consistent expression patterns with the RNA-seq results. This consistency provides a solid foundation for subsequent screening and functional hypothesis of core *PmCCT* genes. This suggests that different *PmCCT* genes may play distinct roles in responding to light signals and regulating spikelet development, indicating that plants may employ tissue-specific regulatory pathways for photoperiod perception and signal transduction.

### 4.5 Ten *PmCCT* genes are candidate markers associated with heading date regulation

By analyzing the expression dynamics of 17 candidate *PmCCT* genes during the critical heading transition period (21D and 28D) under SD conditions, ten *PmCCT* genes (*PmCCT6*, *PmCCT19*, *PmCCT21*, *PmCCT22*, *PmCCT24*, *PmCCT29*, *PmCCT36*, *PmCCT59*, *PmCCT62*, and *PmCCT63*) were identified whose upregulation was specifically associated with the heading event in the photoperiod-sensitive accession Longmi4 (**Figure 8**). Their expression levels remained largely unchanged or decreased in the non-heading, photoperiod-insensitive accessions 0016 and 0053 during the same period. This temporal expression pattern, rather than absolute expression at a single time point, strongly implicates these genes in the heading process, positioning them as promising heading-associated marker genes.

Among these, *PmCCT19*, *PmCCT21*, *PmCCT36*, *PmCCT62*, and *PmCCT63* belong to the CMF subfamily; *PmCCT6*, *PmCCT22*, *PmCCT29* and *PmCCT59* to the COL subfamily; and *PmCCT24* to the TIFY subfamily. The prevalence of CMF subfamily members among these markers is consistent with the known roles of CMF genes like *Ghd7* in rice and *ZmCCT* in maize as central regulators of flowering time (Jin et al., 2018; Liu et al., 2013; Tan et al., 2016). The identification of markers from multiple subfamilies further underscores the complexity of the photoperiodic flowering network in proso millet, where genes from different families may converge to regulate this key agronomic trait. Notably, among these ten markers, only *PmCCT19* was consistently upregulated in both leaves and SAM in response to SD conditions in our initial transcriptome screen (**Figure 6**), suggesting potential functional specialization at the tissue level. These findings provide a refined set of candidate genes for future functional studies aimed at manipulating heading date in proso millet.

In conclusion, this study presents a comprehensive genome-wide analysis of the CCT gene family in proso millet, identifying ten candidate marker genes with expression dynamics tightly linked to heading transition. These findings not only reveal the molecular characteristics and photoperiod-responsive expression patterns of the *PmCCT* gene family but also provide valuable molecular targets for breeding programs aimed at optimizing heading date, improving geographical adaptation, and expanding the cultivation range of proso millet.

## Data Availability

The datasets presented in this study can be found in online repositories. The names of the repository/repositories and accession number(s) can be found in the article/**Supplementary Material**.
