# Supplementary material for: Genome-wide identification and expression analysis of the CCT gene family reveals its function in regulating heading date in proso millet (Panicum miliaceum L.)
Source: Front Plant Sci. 2026 Mar 17;17:1721307. doi: 10.3389/fpls.2026.1721307 (PMC13036209; doi:10.3389/fpls.2026.1721307)
Supplement: Supplementary file 7 [file Table7.docx]

## Supplementary Figures

**
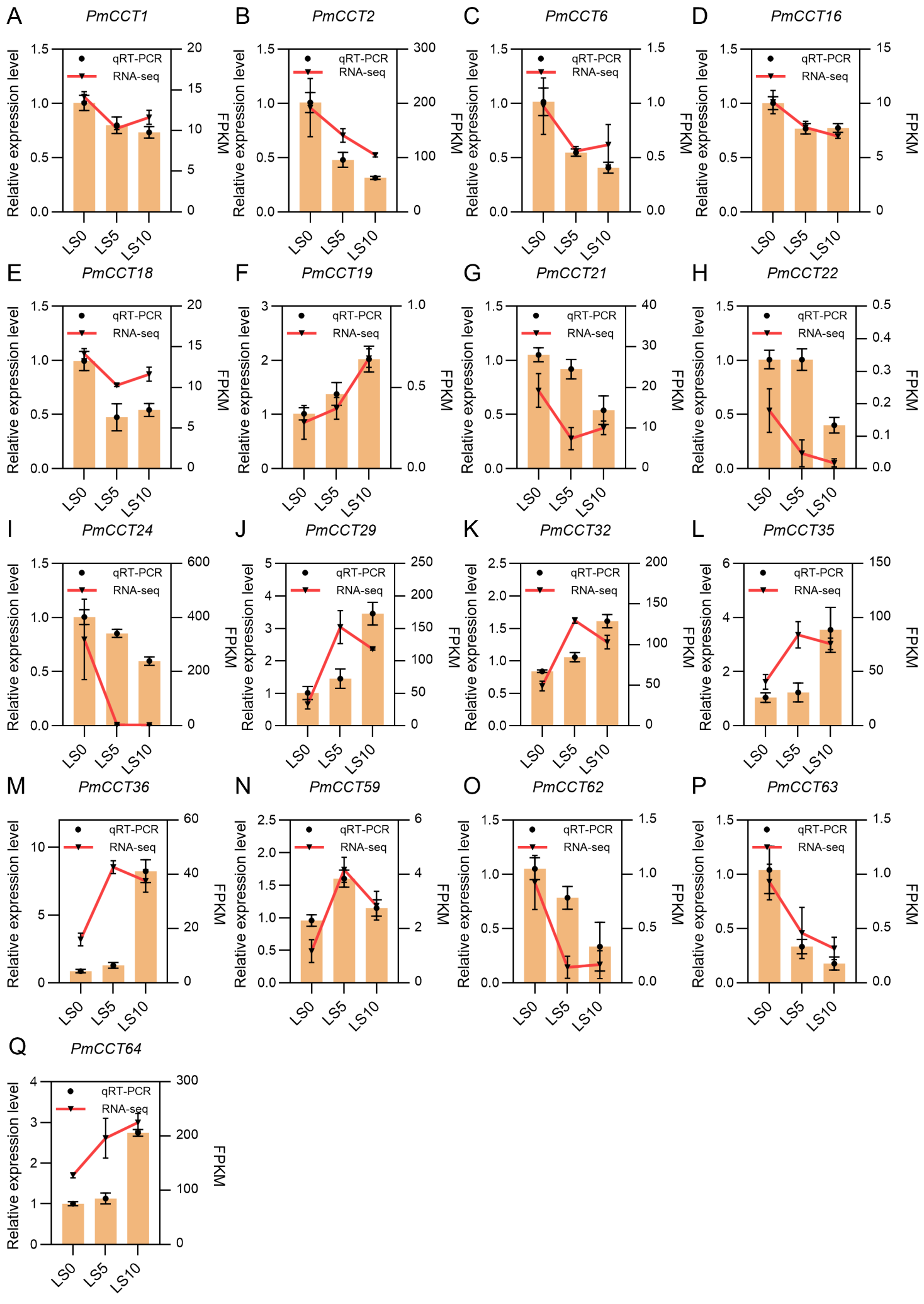
**

**Supplementary Figure 1.**

qRT-PCR validation of 17 *PmCCT* genes in leaves and comparison with transcriptome data. The relative expression levels of 17 *PmCCT* genes in leaves of 'Longmi4' under SD treatment at S0, S5, and S10 time points were detected by qRT-PCR. The left Y-axis represents the relative expression levels of *PmCCT* genes measured by qRT-PCR, and the right Y-axis represents the FPKM values of the corresponding *PmCCT* genes from transcriptome data. Bars represent the qRT-PCR results, with heights indicating the mean expression levels from three independent biological replicates, and error bars indicating the standard error of the mean (SEM). All experiments were repeated at least three times with similar results. The line graph represents the FPKM values from the transcriptome data.

**
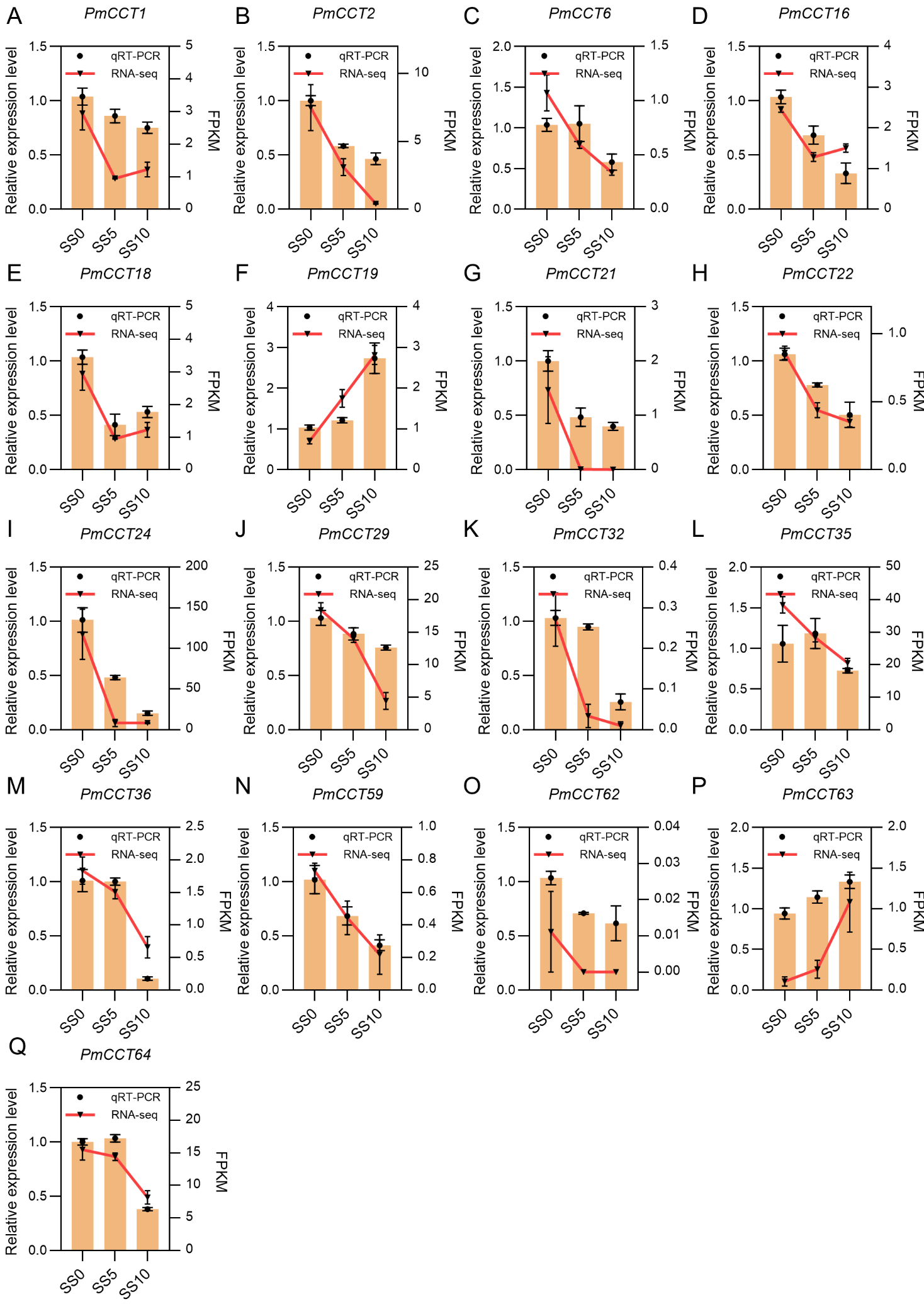
**

**Supplementary Figure 2.**

qRT-PCR validation of 17 *PmCCT* genes in SAM and comparison with transcriptome data. The relative expression levels of 17 *PmCCT* genes in SAM of 'Longmi4' under SD treatment at S0, S5, and S10 time points were detected by qRT-PCR. The left Y-axis represents the relative expression levels of *PmCCT* genes measured by qRT-PCR, and the right Y-axis represents the FPKM values of the corresponding *PmCCT* genes from transcriptome data. Bars represent the qRT-PCR results, with heights indicating the mean expression levels from three independent biological replicates, and error bars indicating the standard error of the mean (SEM). All experiments were repeated at least three times with similar results. The line graph represents the FPKM values from the transcriptome data.
